# An investigation of commercial carbon air cathode structure in ionic liquid based sodium oxygen batteries

**DOI:** 10.1038/s41598-020-63473-y

**Published:** 2020-04-28

**Authors:** The An Ha, Cristina Pozo-Gonzalo, Kate Nairn, Douglas R. MacFarlane, Maria Forsyth, Patrick C. Howlett

**Affiliations:** 1 ARC Centre of Excellence for Electromaterials Science, Institute for Frontier Materials, Deakin University, 221 Burwood Highway, Victoria, 3125 Australia; 2 ARC Centre of Excellence for Electromaterials Science, School of Chemistry, Monash University, Victoria, 3800 Australia

**Keywords:** Energy, Materials for energy and catalysis

## Abstract

In order to bridge the gap between theoretical and practical energy density in sodium oxygen batteries challenges need to be overcome. In this work, four commercial air cathodes were selected, and the impacts of their morphologies, structure and chemistry on their performance with a pyrrolidinium-based ionic liquid electrolyte are evaluated. The highest discharge capacity was found for a cathode with a pore size *ca*. 6 nm; this was over 100 times greater than that delivered by a cathode with a pore size less than 2 nm. The air cathode with the highest specific surface area and the presence of a microporous layer (BC39) exhibited the highest specific capacity (0.53 mAh cm^−2^).

## Introduction

Electric vehicles (EVs) and hybrid electric vehicles (HEVs) are rapidly increasing in popularity^1^. However, the limited specific energy of current energy storage technologies remains a significant issue^2^. Metal oxygen batteries (also known as metal air batteries) are promising candidates for vehicles, owing to their high theoretical energy density^1,3–5^. For instance, lithium oxygen (Li-O_2_) batteries have a theoretical gravimetric energy density of 3456 Wh kg^−1^, assuming lithium peroxide (Li_2_O_2_) as the discharge product. The theoretical gravimetric energy density of sodium oxygen (Na-O_2_) batteries depends on the assumed discharge product, and is 1605 Wh kg^−1^ or 1105 Wh kg^−1^ for sodium peroxide (Na_2_O_2_) or sodium superoxide (NaO_2_), respectively. The attention paid to Na-O_2_ batteries (also known as sodium air batteries) is not just because of their high theoretical energy density, but also because sodium is an abundant resource^5^. The first study of Na-O_2_ batteries was reported in 2011 by Peled *et al*.^6^, and the number of studies has grown rapidly. Compared to Li-O_2_ batteries, Na-O_2_ batteries have lower charge over-potentials^5^.

However, in practice, there remain many challenges — poor capacity, electrolyte degradation, and poor cycling performance^7,8^. In metal oxygen batteries, the air cathode needs to provide enough space to store the products generated during discharge. Otherwise, precipitation of large amounts of insoluble discharge products on the air cathode can block the oxygen-diffusion pathway, resulting in cell failure^9^. The morphology of the discharge products is also critical – an impervious coating of discharge products can passivate the electrode, and drastically reduce cell performance^10^. In addition, the corrosion of carbonaceous air cathodes during battery operation can generate undesired products (Na_2_CO_3_) and increase the overpotential during charge (OER-oxygen evolution reaction)^9,11–14^. Consequently, the efficiencies and cycling performance of sodium batteries is reduced^9,11,12^. Hence, to improve battery performance, it is essential to understand how the structure and properties of the air cathode affect the specific capacity of the battery^5,8^.

To address this challenge, various commercial carbon materials with varying specific surface areas, particle sizes and morphologies were investigated by Bender *et al*.^15^ using 0.5 M sodium trifluoromethanesulfonate in diethylene glycol dimethyl ether (NaOTf/diglyme) as the electrolyte. NaO_2_ was obtained as the main discharge product on all of the cathodes examined (free-standing GDL H 2315, Ketjenblack EC600JD, Super P- Li, HSAG 500, SFG-44 and SCR-1). The discharge capacities of those air cathodes ranged from 0.5 mAh cm^−2^ to 2.9 mAh cm^−2^ at the same applied current (0.2 mA cm^−2^), illustrating the effects of structural differences.

The roles of porosity and specific surface area in Na-O_2_ batteries were also studied using NaOTf in tetraethylene glycol dimethyl ether (tetraglyme) electrolyte^16^. In that study, commercial carbon black was heat-treated under different atmospheres (NH_3_, CO_2_, or CO_2_ with H_2_), for different periods of time, producing carbon mass losses from 10 to 85%; these heat-treated carbons were then used as air cathodes^16^. The specific discharge capacity of the Na-O_2_ batteries increased significantly with carbon mass loss, from 0.13 mAh cm^−2^ to 0.98 mAh cm^−2^ at the same applied current density (0.027 mA cm^−2^). There are several effects to consider. As the carbon mass loss increases, the surface area of the air cathodes also increases; this provides more active sites for the oxygen reduction reaction, consistent with the larger capacity in the battery. In addition, the greater electrode porosity means that more diffusion pathways are available for both oxygen and sodium cations, along with more space to accommodate discharge products, thus further increasing the cell capacity^16^. Significantly, it was concluded that the morphology of the discharge product depends on the specific surface area and pore size of the cathode, while the current density affects the chemical composition of the discharge product.

Yadegari *et al*.^17^ reported the influence of the hydrophobic or hydrophilic character of air cathodes on the morphologies and composition of the reaction products in 0.5 M NaOTf /diglyme. With a hydrophobic air cathode, cubic crystalline sodium superoxide was the sole discharge product; in contrast, amorphous discharge products were found with a more hydrophilic air cathode surface. However, most of the studies on sodium-oxygen batteries use organic solvents in the electrolyte which, due to their volatility, toxicity and flammability, can be expected to present many practical challenges for device applications.

Ionic liquids are an attractive alternative electrolyte because of their high ionic conductivity, good electrochemical stability, wide electrochemical windows and non-flammability^18^. Those properties add to the overall safety of high energy density metal based batteries. Additionally, their negligible volatility and demonstrated ability to perform in the presence of moisture are key features in metal air cells where an open air cathode is necessary^19–22^. Finding electrolytes that are stable and resistant to degradation during battery operation is also critical for metal oxygen batteries, where parasitic side reactions between the electrolyte and active species (such as superoxide) pose a significant challenge^23^. Certain ionic liquids, such as those containing the substituted pyrrolidinium cation have poor leaving groups bound to the N atom, providing stability against alkali metal electrodes and making them less prone to superoxide attack, and hence more resistant to degradation as reported in the literature^24–26^.

Despite this, the use of ionic liquids as electrolytes in Na-O_2_ batteries has rarely been reported^27–32^. Pozo-Gonzalo *et al*.^29,30^ reported the oxygen reduction reaction mechanism (ORR) in *N*-butyl-*N*-methylpyrrolidinium bis(trifluoromethylsulfonyl)imide [C_4_mpyr][TFSI], solutions containing different concentrations of sodium bis(trifluoromethylsulfonyl)imide (NaTFSI), and found that the concentration of NaTFSI affected the ORR mechanism. Later, Zhang *et al*.^28^ studied how the sodium ion concentration in [C_4_mpyr][TFSI] affected the morphology of the discharge products, and how this impacted cell cyclability. They found that the deposit formed with higher concentrations of NaTFSI (*ca*. 0.5 mol kg^−1^) in [C_4_mpyr][TFSI] resulted in limited passivation and far less blockage of the electrode surface than that formed with lower concentrations^28^. In addition, Firth *et al*.^33^ used *in-situ* Raman spectroscopy for a lithium oxygen battery using ([C_4_mpyr][TFSI]) and 1-ethyl-3-methylimidazolium bis(trifluoromethylsulfonyl)amide ([C_2_mim][TFSI]) based ionic liquid and revealed that ([C_4_mpyr][TFSI]) was stable against the superoxide ion during the oxygen reduction reaction, as opposed to the imidazolium analogue.

Zhao *et al*.^31^ reported a Na-O_2_ cell using vertically aligned carbon nanotubes (VACNTs) grown on stainless steel networks as the air cathode and *N*-methyl-*N*-propylpiperidinium bis(trifluoromethylsulfonyl)imide as the electrolyte. Spectroscopic analysis of the cathodes was performed after discharge. Interestingly, most of the “discharge products” obtained — such as sodium hydroxide, sodium carbonate and sodium carboxylates — were identified as originating from side reactions. A weak peak corresponding to sodium superoxide was also detected by XRD. This indicated that *N*-methyl-*N*-propylpiperidinium bis(trifluoromethylsulfonyl)imide was not stable during battery cycling, undergoing parasitic reactions instead of the oxygen reduction reaction.

The hypothesis investigated in this work is that air cathode features will modify the discharge characteristics of Na-O_2_ cells with ionic liquid electrolytes and that this is an important and poorly understood area. Here, we report for the first time a study of different air cathodes in a pyrrolidinium-based ionic liquid electrolyte. We examined the performance of Na-O_2_ cells with several different commercial carbonaceous air cathodes to understand the roles morphology, structure and chemistry on sodium-oxygen battery operation.

We have found a relationship between the specific surface area and the performance of Na-O_2_ cells using a pyrrolidinium based ionic liquid electrolyte. An additional microporous layer and PTFE as hydrophobic character on the air cathode led to increased performance. Thus, this study informs the direction and features that can be employed for optimizing air cathode performance in Na-O_2_ batteries using safe and stable ionic liquid electrolytes.

## Experimental

### Materials

H 23 (Freudenberg) and BC 39 (Sigracet) (with a Microporous Layer (MPL) and 5 wt % PTFE) were purchased from Fuel Cell Store. MGL 190 with 5 wt % PTFE (AvCarb Material Solutions) and Toray 090 (Toray Carbon Paper 90 – TGP-H-090- with a Microporous Layer (MPL) and 25 wt % PTFE) were purchased from Shanghai Hesen Electric Co. Ltd.

*N*-butyl-*N*-methylpyrrolidinium bis(trifluoromethylsulfonyl)imide, [C_4_mpyr][TFSI], (99.9%) and sodium bis(trifluoromethylsulfonyl)imide (NaTFSI) were purchased from Solvionic SA. Both chemicals were opened and stored in an Argon glovebox (Korea Kiyon, with nominal levels of oxygen and water less than 1 ppm).

Tetrahydrofuran (THF, Merck 99%) was dried using activated molecular sieves (4 Å) for one week before use. The water content in the THF was measured by a Metrohm KF 831 Karl-Fischer coulometric titration as <10 ppm. Ultra-high purity oxygen (>99.999% Oxygen, <5 ppm moisture and <3 ppm hydrocarbon gas) was purchased from Supagas.

### Preparation of electrolyte

A 16.6 mol % NaTFSI/[C_4_mpyr][TFSI] electrolyte mixture was prepared by adding NaTFSI (0.4765 grams, 0.0016 mol) into [C_4_mpyr][TFSI] (4 grams, 0.0095 mol) and stirring at room temperature in the Argon glovebox for 24 hours. After that, the electrolyte was dried on a Schlenk line at 50 °C for 24 hours. The water content was <50 ppm, as per Karl-Fischer titration.

### Half-cells

Before use, all the carbon papers were punched into 7 mm diameter discs, washed with ethanol and dried in a vacuum oven for 24 hours at 120 °C, and 12 hours at 150 °C before transferring to the Argon glovebox.

An in-house three-electrode half-cell was used in this study (described previously by Zhang *et al*.^28^), with continuous oxygen flow. In this cell, a platinum wire coil was used as the counter electrode and commercial air cathodes (e.g. H 23, MGL 190, BC39 and Toray 090) with a geometrical area of 0.385 cm^2^ were employed as working electrodes. A reference electrode was prepared by immersing a silver wire in 5 mM silver trifluoromethanesulfonate in [C_4_mpyr][TFSI]. The electrolyte volume was 500 µL. The experiments were performed in a bespoke oxygen/nitrogen glovebox (O_2_/N_2_ glovebox) (from 100 ppm to 250 ppm water).

The reference electrode was calibrated versus ferrocene (E_1/2_ = +0.40 V). All potentials in the discharge and charge curves are reported vs Na/Na^+^ potential (−2.98 V vs Fc^0^/Fc^+^).

### Full cells

To make the Na-O_2_ full cells, CR2032 coin cells were assembled in the Argon glovebox. One of the cases of the coin cell had 19 holes (10 mm diameter), to allow oxygen to flow inside the cell. Sodium metal foil was cleaned inside the Argon glovebox with hexane to remove surface impurities, and then punched into a 10 mm diameter disc. The commercial air cathodes were punched into 12 mm diameter discs (a geometrical area of 1.13 cm^2^). The air cathodes were thoroughly washed, as above, prior to electrochemical characterization. The 16.6 mol % NaTFSI in [C_4_mpyr][TFSI] electrolyte mixture was bubbled with oxygen for 15 minutes; two Celgard 2400 separators (Celgard) were then immersed in the electrolyte for 5 minutes. A further 60–90 µL of electrolyte was added onto the separator when assembling the cell.

The assembled coin cells were placed in a housing cell (Fig. S1) prepared by modifying a Schott Duran bottle with two channels to allows oxygen to flow during the battery operation. The modified Schott Duran bottle was transferred in a canister to a bespoke oxygen/nitrogen glovebox (O_2_/N_2_ glovebox) (from 100 ppm to 250 ppm water).

The oxygen/nitrogen glovebox uses Zero Air grade gas (Supagas), containing only nitrogen and oxygen (from 20% to 23% oxygen in nitrogen) and no carbon dioxide. The cells were flushed with pure oxygen (99.9%) for 5 minutes and rested for 3 hours before cycling. All measurements were carried out on a Biologic VMP3/Z multi-channel potentiostat or Biologic SP-200 potentiostat.

### Characterization of the air cathodes and discharge products

Nitrogen adsorption and desorption tests were performed with liquid nitrogen at 77 K on a Quantachrome Autosorb-IQ MP instrument. Before measurement, the air cathodes were dried as before. After that, the samples were degassed for 24 hours before analysis to remove traces of water and ensure more accurate results. The specific surface area was calculated from the absorption data by the Brunauer−Emmett−Teller (BET) equation. The Barrett−Joyner−Halenda (BJH) procedure was used to calculate the pore size distribution from the nitrogen sorption isotherm.

SEM (Scanning Electron Microscopy) and EDS (Energy Dispersive X-ray Spectroscopy) were used to characterize the morphology and determine the elemental composition of the discharge products. The SEM instrument (JOEL JSM IT300 Oxford) was integrated into the EDS system for element analysis. To characterize the discharge products, the Na-O_2_ battery was first discharged in the O_2_/N_2_ glovebox, then sealed in a canister and transferred to the Argon glovebox. The cell was disassembled and the cathode was washed with dry THF and dried for 5 minutes at room temperature in the argon glovebox, following the procedure previously reported^28^. Then, the cathode was placed in an air-tight holder for transfer into the SEM vacuum chamber. The samples for Raman characterization were also washed by the same procedure and then stored in an airtight sample holder equipped with one microscope slide and a cover slip for laser transmission, with parafilm used to seal between the slide and the cover slip (Fig. S2). Raman spectroscopy was performed with a Renishaw InVia Raman spectrometer at 632 nm, using an air-cooled Argon ion laser for excitation at room temperature through a 50× objective. Before measurement, the spectrometer was calibrated with silicon (520.5 cm^−1^).

## Results and discussion

### Porosity and morphological properties of carbon air cathodes

**Figure 1:**
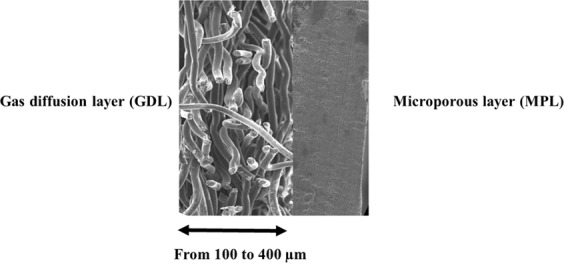
Schematic of the air cathode composition.

The commercial air cathodes selected for Na-O_2_ cell studies had different compositions and structures, as described in Table 1 and Fig. 1. All of the selected cathodes had a gas diffusion layer (GDL), a porous material composed of a dense array of carbon fibres, and in some cases, carbon black. In addition to this, Toray 090 and BC 39 also had a microporous layer (MPL); these MPLs were composed mostly of carbon black, with different PTFE contents (25% wt for Toray 090 and 5% wt for BC 39) (Fig. 1). It is important to highlight that these air cathodes have different thicknesses and masses (Table 1); with those containing the microporous layer being thicker and heavier.

**Table 1:** Summary of the structures and compositions of the air cathodes under study.

	H 23	MGL 190	Toray 090	BC 39
Microporous layer (MPL)	None	None	One side	One side
PTFE content (as reported by manufacturers) (wt %)	0%	5%	25%	5%
Thickness (mm)	0.20 ± 0.01	0.19 ± 0.02	0.28 ± 0.01	0.33 ± 0.01
Thickness of MPL (mm)	None	None	0.08 ± 0.01	0.10 ± 0.01
Mass (of a 12 mm diameter circle) (mg)	9.0 ± 0.1	9.3 ± 0.1	16.0 ± 0.1	9.8 ± 0.1
Specific surface area (m^2^ g^−1^)	Below 1	5.6	11.3	22.5

**Figure 2:**
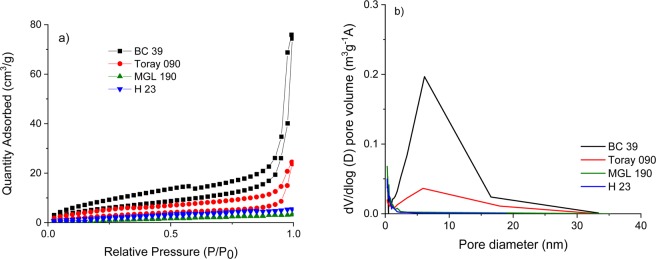
(**a**) Nitrogen sorption isotherms and (**b**) pore size distribution corresponding to BC 39, Toray 090, MGL 190 and H 23.

To determine how the MPL affects the specific surface area and pore size distribution, nitrogen sorption isotherms over a relative pressure (P/P_0_) range from 0 to 1.0 were measured (Table 1 and Fig. 2).

H 23 and MGL 190 gave IUPAC type I isotherms^34^, characteristic of microporous carbon. In more detail, an increasing absorbed nitrogen volume is observed at P/P_0_ around 0 and is dominant in a range below P/P_0_ < 0.4. The average pore sizes for H 23 and MGL 190 are below 2 nm, as displayed in the inset to Fig. 2b, and these cathodes also show low specific surface areas (less than 1 m^2^ g^−1^ and 5.6 m^2^ g^−1^ for H23 and MGL 190, respectively).

The isotherms obtained for BC 39 and Toray 090 were IUPAC^34^ type IV, showing an increase in absorbed nitrogen volume at relative pressures from 0.4 to 0.8. This profile is consistent with a pore size around 6 nm (Fig. 2b); these samples also gave larger specific surface areas.

The samples that have the MPL, BC 39 and Toray 090, showed larger specific surface areas than those for H23 and MGL 190. This implies that the carbon black, the main component of the MPL, is contributing to the porosity. This is in agreement with the work reported by Jayakumar and co-workers^35^.

Interestingly, even though BC 39 and Toray 090 share similar composition, thickness of MPL and pore size, the specific surface area of BC 39 is twice that of Toray 090 (22.5 m^2^ g^−1^ vs 11.3 m^2^ g^−1^); this difference may be related to the different PTFE content in the two materials. In fact, Simon *et al*.^36^ reported a decrease in specific surface area of air cathodes by half when increasing the PTFE content from 22% to 41% wt, and attributed this to blockage of the pores in the cathode. Lower content of PTFE in air cathodes (ca. 5% wt) led to the best performance in Li-O_2_ cells when studying a wide range PTFE content from 5% to 35% wt, according to Wang *et al*.^37^. However, it is important to highlight that those works focus on fuel cells and Li-O_2_ cells, while no work on the role of PTFE content on Na-O_2_ cells performance has been previously reported.

**Figure 3:**
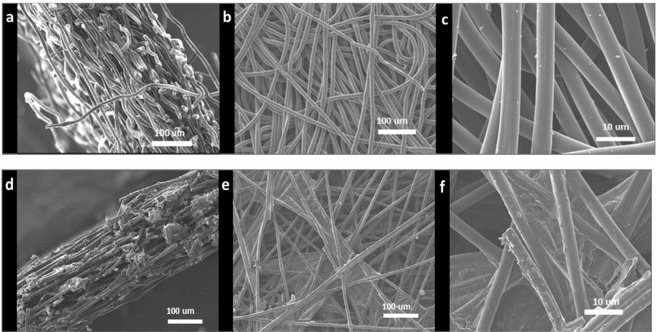
SEM images of H 23 (**a**–**c**) and MGL 190 (**d**–**f**) air cathodes used in this study. Cross sections (**a,b**), and gas diffusion layers at low (**c,d**) and higher (**e,f**) magnifications.

**Figure 4:**
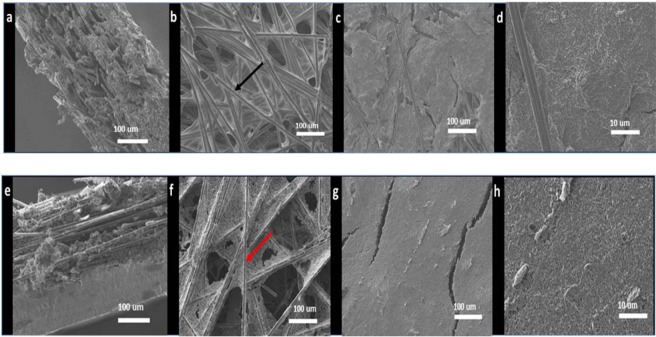
SEM images of Toray 090 (**a**–**d**) and BC 39 (**e**–**h**) corresponding to the cross section (**a,e**), gas diffusion layer (**b,f**), MPL (**c,g**), and MPL at higher magnification showing fibre size (**d,h**).

Scanning electron microscope (SEM) images of the faces and cross sections of the air cathodes are presented in Figs. 3 and 4. H 23 and MGL 190 both show a GDL of non-woven carbon fibres. The fibres in H 23 are ~7–8 micrometres in diameter while those in MPL 190 are 6–7 micrometres in diameter (Fig. 4). Neither of these cathodes contains an appreciable amount of carbon black.

On the other hand, the cross sections of the BC 39 and Toray 090 air cathodes (Fig. 4) show an MPL as well as a GDL. In the case of the Toray 090 electrode, the carbon fibres are covered with carbon black on the MPL side and with PTFE on the GDL side. However, both carbon black and PTFE are present on both sides of BC 39.

Furthermore, more PTFE can be observed on the surface of the GDL for Toray 090 (highlighted with a black arrow, Fig. 4b); in contrast, for BC 39 (highlighted with a red arrow Fig. 4f), carbon black is the main component on the fibre surface.

### Electrochemical properties of carbon papers as air cathodes: half cell

**Figure 5:**
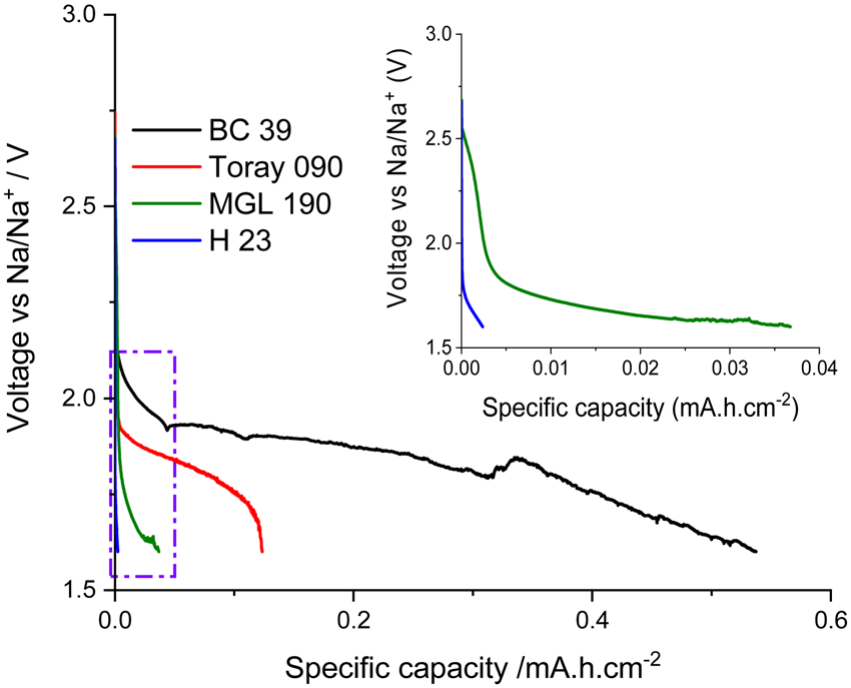
Discharge curves for cells containing the different cathode materials, obtained using a homemade three-electrode half-cell. Applied current 0.623 mA cm^−2^. Cut-off potential: 1.6 V.

To investigate the electrocatalytic properties of these commercial air cathodes, galvanostatic discharge experiments were performed, using a homemade three-electrode half-cell (Fig. 5).

Figure 5a compares the discharge capacities of the four air cathodes using the NaTFSI/[C_4_mpyr][TFSI] IL. The discharge capacity values for H 23 and MGL 190 were 0.0024 mAh cm^−2^ and 0.037 mAh cm^−2^, respectively (Fig. 5a and inset). Interestingly, MGL 190 delivers fifteen times higher specific capacity than H 23. Both these materials have similar pore sizes (below 2 nm); however, they have different specific surface areas (below 1 m^2^ g^−1^ for H 23 and 5.6 m^2^ g^−1^ for MGL 190). An electrode surface with high specific area provides more sites for the oxygen reduction reaction to occur; and is thus expected to increase specific capacity.

When comparing the two MPL-containing air cathodes, BC 39 delivered four times the discharge capacity of Toray 090 (e.g. 0.53 mAh cm^−2^ vs 0.13 mAh cm^−2^, respectively) at the current rate of 0.623 mA cm^−2^ (Fig. 5a). This substantial difference in performance may be related to the different surface areas and pore distribution of both air cathodes. Figure 2b illustrates both air cathodes BC 39 and Toray 090 have the pore distribution (around 6 nm); however, the pore distribution of BC 39 is larger than those of Toray 090. As a consequence, BC 39 provides more active sites for the ORR and more space for storing discharge products, resulting in much larger specific capacity.

Comparing the air cathodes with and without a MPL, the air cathode with a MPL delivered higher capacity. According to the literature^35^, the MPL plays an important role in promoting the ORR and OER processes by increasing the specific surface area. Additionally, mesoporous air cathodes can lead to better performance as reported for lithium oxygen cells by Xiao *et al*.^38^.

Similarly, Ortiz-Vitoriano *et al*.^39^ found the same trend with graphene aerogel cathodes for Na-O_2_ batteries and reported the correlation between pore size distribution (micro vs mesoporous) and specific capacity. However, the specific surface area of the aerogels was not considered in their study, making it difficult to compare their results with the current work.

Finally, it is important to note that these previous reports in the literature focus on the relationships between performance and composition and structure of air cathodes using aqueous or conventional non-aqueous media and in fact there are no reports investigating the role of air cathode structure and composition in IL electrolytes. A variety of factors impact the discharge capacity of sodium oxygen batteries including specific area, pore size, presence of a microporous layer and pore distribution. BC 39, with the presence of a MPL, showed the highest discharge capacity due to its higher specific area, mesopore size range and larger pore distribution. The results in the present work suggest that similar trends in the effect of air cathode morphology are obtained when using ionic liquid electrolytes as with conventional electrolyte systems.

### Morphologies discharge products on carbon papers as air cathodes in the presence of sodium anode

Coin cells were also prepared to investigate the effect of the commercial carbon papers as air cathodes on the anode surface (Fig. S3).

None of the cells could sustain a current density of 0.623 mA cm^−2^ and therefore a lower value of current density (0.24 mA cm^−2^) was applied in this part of the study and cut off potential 0.9 V (Fig. S3). This current is still higher than those used in a study by Zhao and Gou^24^ (0.24 mA cm^−2^ and 0.05 mA cm^−2^, respectively).

**Figure 6:**
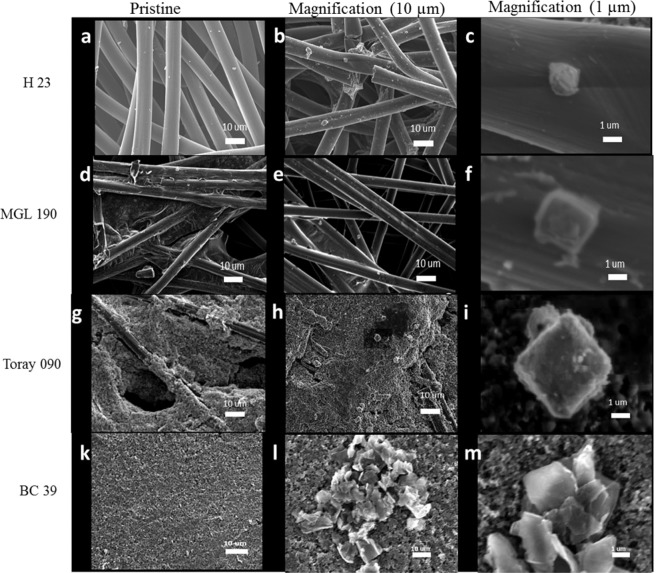
SEM images of the pristine and discharge air cathodes (at 0.24 mAh cm^−2^ and cut off potential at 0.9 V) at two magnifications corresponding to H 23 (**a**–**c**); MGL 190 (**d**–**f**); Toray 090 (**g**–**i**) and BC 39 (**k**–**m**).

SEM images of discharge products within the air cathodes are shown in Fig. 6. The discharge products were cubic, for all air cathodes; however, the particle size and amount of discharge product differed depending on the air cathode.

The discharge products in the air cathodes with smaller pores (H 23 and MGL 190) were 1–2 μm across (Fig. 6c,f). In contrast, the cathodes with the larger pores and microporous layer (Toray 090 and BC 39) gave larger discharge particles, 2–4 μm across (Fig. 6l,m).

Currently, there are almost no studies that correlate the specific surface area of the air cathode with the size of the discharge products. Only Sun’s group[11] have reported that the morphology of the discharge products was dependent on the specific surface area, varying from rod-shaped particles to thin film structures upon increasing the specific surface area. However, in our study the size of the discharge product increases with the specific surface area while the morphology (cubic structure) remains. It is important to highlight that there are significant differences between both studies that could affect the final outcome, such as the nature of the electrolyte (ionic liquid in comparison with tetraglyme), and also the nature and composition of the air cathode.

### Full cell containing BC 39 air cathode

Because BC 39 gave the greatest discharge capacity in the half cell experiments, it was studied in more detail. In this case, the coin cell was discharged at 0.05 mA cm^−2^, which is the same current applied reported in the literature for Na oxygen batteries using ionic liquid as electrolyte^31^.

**Figure 7:**
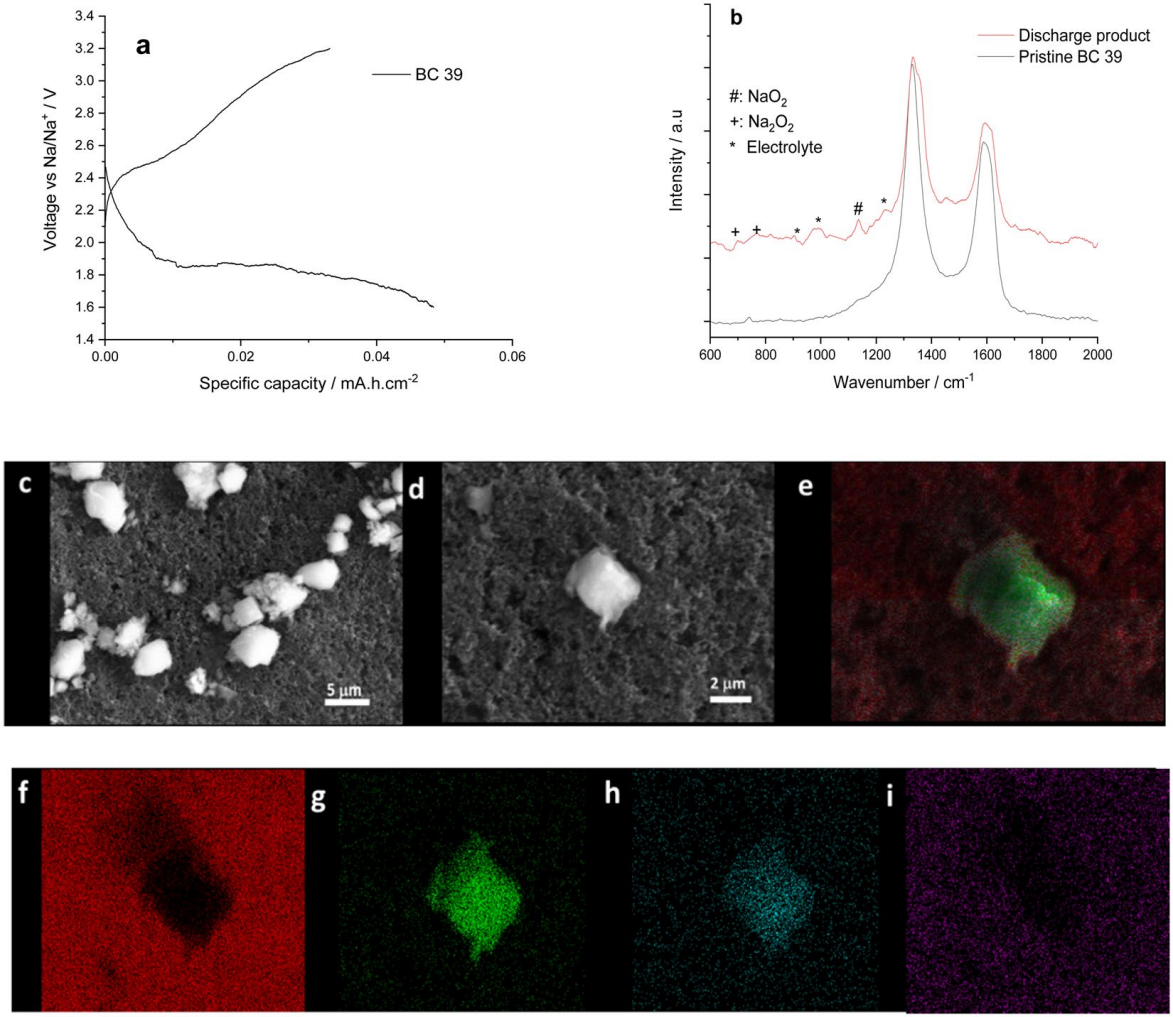
(**a**) The discharge and charge curve of BC 39 (coin cell), (**b**) Raman spectra of pristine BC 39, control sample and BC 39 after first discharge at 0.05 mA cm^−2^; wavenumber range from 600 to 2000 cm^−1^, SEM images of BC 39 after the first discharge scans with magnifications (**c**) 5 µm scale-bar, and (**d**) 2 µm scale-bar; (**e**) EDS mapping for the discharge products in BC 39 and the different elements individually (**f**) carbon, (**g**) oxygen, (**h**) sodium and (**i**) fluorine Applied current 0.05 mA cm^−2^. Cut off potential: 1.6 V to 3.2 V.

The specific capacities for the first discharge (0.49 mAh cm^−2^) and charge processes (0.35 mAh cm^−2^) with efficiencies of 70% are shown in Fig. 7a. During the charge process, there were two regions from 2.40 to 2.70 V and another ranging from 2.70 to 3.20 V probably due to the oxidation of sodium peroxide and superoxide. Yadegari *et al*.^40^ reported similar performance which was associated to the oxidation of a mixture of sodium oxides. SEM-EDX and Raman spectroscopy were used to understand the morphologies of discharge products and their chemical composition after the 1^st^ discharge cycle at 0.05 mAh cm^−2^. Micro cubic particles (around 2–6 µm) and other shaped particles (below 1 µm) were observed on the surface of this air cathode (Fig. 7c,d), similar to those seen in our previous work using Toray 090 air cathodes with the same electrolyte mixture^41^.

EDS mapping of the BC 39 air cathode surface shows oxygen, sodium, carbon and fluorine (Fig. 7f–i); however, the cubic particles contain only sodium and oxygen. This suggests that the fluorine present is due to the PTFE in the air cathode, and not a consequence of a side reaction between the IL and the BC 39.

Raman spectroscopy was used to determine the nature of the discharge product (Fig. 7j). To identify possible signals from the air cathode or ionic liquid electrolyte, a BC39 sample immersed in the 16.6 mol % NaTFSI/[C_4_mpr][TFSI] electrolyte and then washed with dry THF (“control sample”) and a pristine BC39 sample were also characterised.

The black spectrum in Fig. 7b corresponds to the pristine BC 39, and shows two well-defined peaks at 1325 cm^−1^ (D band) and at 1580 cm^−1^ (G band), which are assigned to the vibration of sp^2^ bonds between carbon atoms. The Raman spectrum corresponding to the discharge product (Fig. 7b, red spectrum) present extra peaks, apart from the D and G bands assigned to BC 39. The peaks located at 893 cm^−1^, 997 cm^−1^ and 1250 cm^−1^ (* symbol) belong to 16.6 mol % [NaTFSI]/[C_4_mpyr][TFSI]^42^. The standard Raman signals for Na_2_O_2_ are at 735 cm^−1^ and 791 cm^−1^, according to the literature^7,43^. However, in our study, those values are slightly shifted to 730 cm^−1^ and 790 cm^−1^ (+symbol). Based on the SEM images in Fig. 7c,d), we suggest that the particles below 1 µm in size are probably sodium peroxide. The formation of sodium peroxide as discharge products could cause the high over potential in the first cycle due to the lower electronic conductivity values (e.g. 10^−20^ S cm^−1^). Additionally, a strong peak (# symbol) at 1160 cm^−1^ is observed, which could be related to the formation of sodium superoxide (NaO_2_). The values corresponding to NaO_2_ in the literature range from 1150 to 1158 cm^−1^ when using organic electrolytes^16,43,44^. In addition, Pozo-Gonzalo *et al*.^30^ explored the ORR mechanism in 16.6 mol % NaTFSI/[C_4_mpyr][TFSI], using a pressure cell, and confirmed that a 2-electron process occurs, leading to the formation of Na_2_O_2_ as final product, via NaO_2_. Therefore, this study confirms the existence of both products and hence the ORR mechanism we published previously.

It is interesting to compare the nature of the discharge products in our studies with those found previously for state-of-the-art Na-O_2_ batteries with ionic liquid electrolytes. For instance, the discharge products identified in Na–O_2_ batteries using *N*-methyl-*N*-propylpiperidinium bis(trifluoromethylsulfonyl)imide electrolytes were only composed of side products such as NaOH, sodium carbonates and carboxylates; neither NaO_2_ nor Na_2_O_2_ were identified by Raman or FTIR^31^. On the other hand, Azaceta and co-authors^27,45–47^ reported a mixture of sodium oxides (sodium superoxide and sodium peroxide) and a sodium fluoride side product by X-ray photoelectron spectroscopy, using the same electrolyte mixture (16.6 mol% NaTFSI/[C_4_mpyr][TFSI]). None of these side products have been observed in our study using Raman spectroscopy; the origin of these discrepancies are not clear. One possibility could be related to the applied potential (e.g. 1.6 V and 1.0 V vs Na/Na^+^) which could lead to the ionic liquid degradation. Additionally, different cell design, cathode structure and composition could have a role on the dissimilar results.

More studies focusing on cell design are required to overcome the side reactions occurring on the sodium anode surface.

## Conclusion

The electrochemical properties and performance of four different air cathodes in ionic liquid based sodium oxygen (Na-O_2_) cells were examined, focusing on the roles of the cathode morphology, structure and composition. Cathodes with a microporous layer (MPL) and hydrophobic character gave higher discharge capacity in both half cells and full Na-O_2_ cells employing a 16.6 mol % [NaTFSI]/[C_4_mpyr][TFSI] electrolyte. These effects were explained by a combination of maximizing gas diffusion while accommodating discharge products within the cathode, and maintaining active cathode surface area for reactions.

The presence of an MPL on the air cathode contributes to increase the specific discharge capacity, and this increase in capacity. This is translated to larger specific surface area and therefore a larger amount of active sites for the cathodic process.

These results emphasize that achieving higher specific capacity of a Na−O_2_ battery through using an ionic-liquid based electrolyte relies strongly on optimization of the morphology, structure and composition of the air cathode.

Hence, this study provides a promising avenue to improve performance of sodium oxygen batteries through the optimization of the porosity and hydrophobicity of the air cathodes.

## Supplementary information

Supplementary file.
