# Supplementary material for: An investigation of commercial carbon air cathode structure in ionic liquid based sodium oxygen batteries
Source: Sci Rep. 2020 Apr 28;10:7123. doi: 10.1038/s41598-020-63473-y (PMC7188893; doi:10.1038/s41598-020-63473-y)
Supplement: Supplementary file 1 — Supplementary file. [file 41598_2020_63473_MOESM1_ESM.docx]

**Supporting information**

**An investigation of commercial carbon air cathode structure in ionic liquid based sodium oxygen batteries**

The An Ha^1^, Cristina Pozo-Gonzalo^1*^, Kate Nairn^2^ Douglas R. MacFarlane^2^, Maria Forsyth^1^, Patrick C. Howlett^1*^

^1^ ARC Centre of Excellence for Electromaterials Science, Institute for Frontier Materials, Deakin University, 221 Burwood Highway, Victoria, 3125, Australia

^2^ ARC Centre of Excellence for Electromaterials Science, School of Chemistry, Monash University, Victoria 3800, Australia

^*^Corresponding author

Email: patrick.howlett@deakin.edu.au


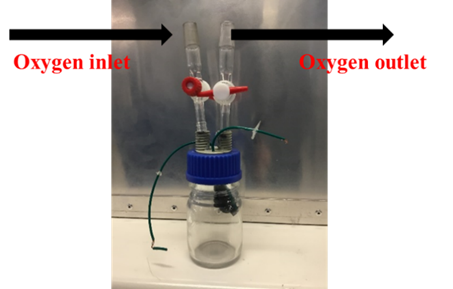


Fig.S 1. An image of housing for coin cell under static condition with two channels for oxygen inlet and oxygen outlet to control the oxygen flow.


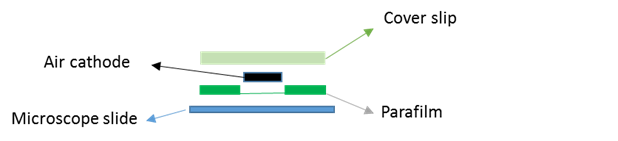


Fig.S 2. A schematic of the airtight sample holder used for Raman spectroscopy of the air cathodes.

Fig. S 3 Discharge curves for cells containing the different cathode materials, obtained using: a coin cell. Applied current 0.24 mA cm^-2^. Cut-off potential: 0.9 V.
